# Growth Factors and Anticatabolic Substances for Prevention and Management of Intervertebral Disc Degeneration

**DOI:** 10.1155/2012/897183

**Published:** 2011-11-03

**Authors:** Umile Giuseppe Longo, Stefano Petrillo, Edoardo Franceschetti, Nicola Maffulli, Vincenzo Denaro

**Affiliations:** ^1^Department of Orthopaedic and Trauma Surgery, Campus Bio-Medico, University of Rome, Via Álvaro del Portillo 200, 00128 Rome, Italy; ^2^Centro Integrato di Ricerca (CIR), Università Campus Bio-Medico di Roma, Via Álvaro del Portillo 21, 00128 Rome, Italy; ^3^Centre for Sports and Exercise Medicine, Barts and The London School of Medicine and Dentistry, Mile End Hospital, 275 Bancroft Road, London E1 4DG, UK

## Abstract

Intervertebral disc (IVD) degeneration is frequent, appearing from the second decade of life and progressing with age. Conservative management often fails, and patients with IVD degeneration may need surgical intervention. Several treatment strategies have been proposed, although only surgical discectomy and arthrodesis have been proved to be predictably effective. Biological strategies aim to prevent and manage IVD degeneration, improving the function and anabolic and reparative capabilities of the nucleus pulposus and annulus fibrosus cells and inhibiting matrix degradation. At present, clinical applications are still in their infancy. Further studies are required to clarify the role of growth factors and anticatabolic substances for prevention and management of intervertebral disc degeneration.

## 1. Introduction

The physiological homeostasis of the various tissues of the intervertebral disc (IVD) is regulated by the active maintenance of the balance between the anabolism and catabolism of IVD cells [1, 2]. This mechanism is maintained through a complex coordination of a variety of substances and molecules, including growth factors, enzymes, enzyme inhibitors and cytokines, that act in a paracrine or/and autocrine fashion [1, 2]. IVD degeneration usually begins from the second decade of life, and progresses with aging [3]. The lack of nutrients [4] and inappropriate mechanical loads [5] may result in loss, alteration, and dysfunction of cell viability and IVD properties [6, 7]. Medical conditions associated with symptomatic IVD degeneration include IVD herniation, radiculopathy, myelopathy, spinal stenosis, instability- and low-back pain, and they represent the most common diagnoses facing spine specialists [8–14]. The accepted treatment for IVD degeneration consists discectomy with vertebral fusion. New biological strategies have not yet been proven to be effective. 

This review reports the state-of-the-art on the management of IVD degeneration using growth factors and anticatabolic molecules.

## 2. Biology of the Intervertebral Disc

The IVD is constituted by three parts: the annnulus fibrosus (AF), the nucleus pulposus (NP), and the endplate (EP). The IVD matrix is composed of an ordered framework of macromolecules able to attract and hold water; the most represented structural components are collagens and proteoglycans [15]. Collagenous proteins are present in the AF while proteoglycans are present in the NP. The function of collagen is to provide form and tensile strength while proteoglycans are responsible for tissue viscoelasticity, stiffness, and resistance to compression through their peculiar interaction with water [6]. Only 20% of collagenous proteins are found in the central NP while a 50% of proteoglycans are located in AF and NP, respectively [15]. The integrity of the IVD is maintained by the balance between matrix synthesis/apposition and degradation. Integrity is maintained by a fine balance of the activity of cytokines, growth factors, enzymes, and enzyme inhibitors, in a paracrine or/and autocrine fashion. Morphological and molecular changes occur in the IVD with aging, determining the progressive degeneration and pathologic alteration of this particular tissue [16]. Morphological changes include dehydratation and tears of the AF, NP, and EPs [16]. Common molecular changes are decreased cell viability and diffusion of nutrient and proteoglycans synthesis, accumulation and increasing of degradative enzymes and degraded matrix macromolecules, and, finally, alteration in collagen distribution [16].

The anabolic function of various growth factors is explained with the accumulation and synthesis of matrix while cytokines demonstrated the opposite effect. They promote catabolism and inhibit synthesis of IVD matrix. 

Several inflammatory mediators have been found in degenerated IVDs, but the real pathologic role of these mediators is unknown or not clearly defined. Nitric oxide (NO), interleukin-6 (IL-6), prostaglandin E2 (PGE2), TNF-alpha, fibronectin, and matrix metalloproteinases (MMPs) are some of the several mediators identified [16–20]. IL-6, NO, and PGE2 have been proposed to be the inhibitory factors of proteoglycan synthesis. These factors are recruited into action by interleukin-1 (IL-1), which also plays a role in the direct degradation of the proteoglycan matrix. This process of direct breakdown by IL-1 is thought to be mediated by a family of enzymes known as MMPs. IL-1 likely plays a major role in the cascade of inflammatory mediators, but the nature of that role is not well defined [21], suggesting that the identification of all mediators that promote degradation of the IVD or accumulation of matrix should be investigated to explore new therapy strategies.

## 3. Biological Therapy Strategies

Therapeutic strategies under investigation for the biological treatment of IVD degeneration include the use of cellular components (mesenchymal stem cells, chondrocytes, disc allograft, culture expanded, disc cells, etc.), molecules influencing disc-cell metabolism and phenotype and matrix-derivatives [22, 23]. The rationale of the biological strategies for arresting and preventing IVD degeneration is linked with the possibility to improve the accumulation of ECM by promoting its synthesis and/or inhibiting its degradation.

This is also connected with IVD biological properties: cells of the AF and NP respond to a different number of cytokines. In fact, IVD degeneration is associated with reduced cellularity, and restoration may be aided by treatments that protect against cell death and apoptosis, or promote mitosis.

Several growth factors, including bone morphogenetic protein-2 (BMP-2), BMP-7 (also known as osteogenic protein-1 [OP-1; Stryker, Kalamazoo, Michigan]), growth and differentiation factor-5 (GDF-5), transforming growth factor-*β* (TGF-*β*), and insulin-like growth factor-1 (IGF-1) stimulate matrix production while interleukin-1 (IL-1) and tumor necrosis factor (TNF) inhibit the synthesis of matrix and enhance its catabolism.

### 3.1. Enzymatic and Nonenzymatic Mechanisms

Other target candidates of therapy include enzymatic and nonenzymatic mechanisms involved in reabsorbing and degrading the IVD matrix. Various types of proteinases, such as matrix metalloproteinases, cathepsins, and members of the ADAM (a disintegrin and metalloprotease) family, are implicated in IVD matrix degradation. Nonenzymic mechanisms involving molecules such as nitric oxide, peroxynitrite, and superoxide are also candidates, but the extent to which they are restrained by the anoxic environment of the IVD is unknown. Most of these experimental solutions have some *in vitro* data, but only few studies have been extended from *in vitro* observation to an animal model of IVD degeneration, especially to large animals models that can be compared to human IVD disease. 

In addition, as several animals were used to study IVD degeneration, including chemical and nonchemical strategies to simulate IVD degeneration *in vitro* and *in vivo*, there is no agreement on which model best simulates human IVD degeneration.

### 3.2. Growth Factors

Growth factors (GFs) are fundamental components for the biological homeostasis of the IVD tissues, and are also fundamental for the development of the spine. Several GFs have been found in degenerated and normal IVD tissues, suggesting that IVD cells are capable of expressing and producing GF and to respond to GFs stimulation, changing their phenotype according to the GFs present at a given time. Indeed, stimulation of matrix synthesis by GFs alters the tissue homeostasis and changes cellular metabolism to the anabolic state [2].

The most studied GFs in IVD degeneration are TGF-b1-3 [24–26], IGF-1 [25, 27], basic fibroblast growth factor (bFGF) [28–30], growth differentiation factor-5 (GDF-5) [31], platelet derived growth factor (PDGF) [32], BMP-2 [33], and BMP-4 [31, 33]. 

TGF-b1 and -b2 and bFGF were found to be expressed in human degenerated IVDs [34]. TGF-b receptor II (TGF-bRII), FGF receptor 3, and BMPRII were also found to be present to a similar extent in both human diseased and normal tissues [35]. 

The rationale for the use of GFs for prevention and treatment of IVD degeneration lies in their capacity to regulate phenotype of IVD cells, promoting an anabolic state with enhanced matrix synthesis and accumulation.

### 3.3. *In Vitro * Studies on Growth Factors Application

Cell proliferation and matrix synthesis and metabolism can be upregulated when exogenous growth factors are applied to tissue or cell cultures.

In 1991, Thompson et al. [36] first described the effects of various growth factors, including TGF-b, epidermal growth factor (EGF), and bFGF on proteoglycan (PG) synthesis by IVD cells. TGF-b1 was expressed frequently by NP cells and occasionally by AF cells when they were spatially associated with fibronectin-synthesizing cells [37]. Moreover, in human three-dimensional IVD cell cultures, TGFb stimulates cell proliferation of annulus cells after four days of exposure [38]. IGF-1 stimulated PG synthesis by bovine NP cells in serum-free conditions in a dose-dependent manner [27].

In addition, IGF-1 and PDGF significantly reduced the percentage of apoptotic AF cells induced by serum depletion in culture [39] while, in rabbit IVD cells, the responsiveness of IVD cells to IGF-1 and TGF-b decreases with increasing age [40]. Melrose et al. [29] showed that, in monolayer cultures of rat AF cells, recombinant human BMP-2 (10–1000 ng/mL) increased cell proliferation, mRNA expression of collagen type II, aggrecan, osteocalcin, and SOX9 and PG synthesis Yoon et al. [41]. Another study reported that BMP-2 significantly increased the expression of aggrecan, collagen type II, TGFb1, and BMP-7 mRNA, and, on the other hand, downregulated versican gene expression [40]. BMP-2 increases and facilitates the expression of the chondrogenic phenotype by human IVD cells. rhBMP-2 increased PG synthesis and upregulated the expression of aggrecan, collagen type I, and collagen type II mRNA, compared to untreated control levels [42]. In rabbit IVD cells, BMP-7, also known as osteogenic protein-1 (OP-1) strongly stimulates the production and formation of PG and collagen and slightly affected cell proliferation [43]. 

In both NP and AF cells, recombinant human OP-1 (rhOP-1) stimulated the production and accumulation of PG and collagen in a dose-dependent manner (50–200 ng/mL) in the presence of 10% fetal bovine serum (FBS). Data from previous cadaveric studies indicated that the capacity of NP and AF cells to synthesize PGs decreases with age [44].

However, OP-1 significantly stimulated PG synthesis in all fetal, adult, and old bovine NP and AF cells [45], suggesting that the IVD cells in older animals are responsive to GFs, such as OP-1.

OP-1 (100–200 ng/mL) improved the *in vitro* production of PGs by human NP and AF cells cultured in alginate beads, especially in the presence of 10% FBS [43]. 

OP-1 also enhanced the accumulation of PGs in the matrix, and AF cells, which are more fibrochondrocytic, strongly responded to OP-1: OP-1 might be beneficial not only for nucleus repair but for anular repair too [43]. Takegami et al. treated the IVD cells cultured in alginate beads with IL-1 [46] or chondroitinase ABC (C-ABC) [47] to damage or to deplete the PG-rich matrices, simulating IVD degeneration. After depletion of the ECM following exposure of IVD cells to IL-1, OP-1 was found to be effective in the replenishment of a matrix rich in PG and collagen. 

OP-1 treatment increased DNA content, and collagen and PG synthesis and accumulation in both early and advanced stages of IVD degeneration, but to a greater and successful extent in early stages of IVD degeneration [48]. The use of growth factors may be suitable in the early rather than in the late stages of IVD degeneration.

Growth and differentiation factor-5 (GDF-5), another member of the BMP family, was firstly described in the literature for its capacity to be a crucial factor responsible for skeletal alterations in brachypodism mice [49]. GDF-5-deficient mice showed IVD degradation and degeneration, and with a low T2-weighted signal intensity and loss of normal lamellar architecture of the AF at MRO, and disorganized NP with a decreased PG content [50]. GDF-5 stimulated collagen type II and aggrecan expression in mouse IVD cells [50].

Some studies also indicated that rhGDF-5 promoted matrix synthesis/accumulation and cell proliferation by both bovine NP and AF cells, with a better response by NP cells compared with AF cells [45].

Platelet-rich plasma (PRP) is a plasma fraction that contains multiple growth factors concentrated at high levels [48, 51, 52], and is produced by the centrifugation of the blood [53, 54]. PRP, administered in porcine cells cultured in alginate beads, is an effective stimulator of cell proliferation and PG and collagen synthesis, as well as PG production and accumulation, by both AF and NP cells [55].

The combination of autologous IL-1 receptor antagonist (IL-1ra)/IGF-1/PDGF proteins reduced the percentage of cellular apoptosis and the production of biochemical markers of IVD degeneration, such as IL-1 and IL-6. These results suggested the possibility of new strategies, using an autologous protein mixture, containing IL-1ra/IGF-1/PDGF, for the treatment of degenerative IVD disease [56].

### 3.4. *In Vivo * Studies on Growth Factors Application

The first study on GF injection into the IVD was reported by Walsh et al. [57] in the mouse caudal IVD degeneration model. In this study, IVD degeneration was induced by static compression [57]. A single injection of GDF-5, but not that of IGF-1, TGF-b, or bFGF, promoted IVD regeneration while multiple injections (four injections, one per week) of TGF-b showed a stimulatory effect. Finally, multiple injections of IGF-1, GDF-5, and bFGF did not show a significant enhancement of their primary effect [57]. A combined approach with a mechanical or cell-based device or a sustained delivery system was required to obtain a beneficial therapeutic effect.

An et al. [58] performed an *in vivo* experiment using the normal rabbit IVD and a single injection of OP-1 (2 lg per disc) to test the efficiency of OP-1 for the treatment of degenerative IVD disease. A single *in vivo* intradiscal injection of OP-1 resulted in increased PG content of the NP and disc height (15%) [58].

Additionally, the same authors conducted a study of radiographic and magnetic resonance imaging findings in the rabbit IVD after injection of OP-1 into the NP in the anular-puncture disc degeneration model. IVD degeneration was induced by anular puncture in two noncontiguous discs with an 18 G needle [59]. Four weeks after the anular puncture, either 5% lactose or OP-1 was injected into the center of the NP. Six weeks after the intradiscal administration of OP-1, an increase of signal intensity of NP in T2-MRI associated with restoration of disc height were observed and sustained along the entire experimental period, up to 24 weeks [60]. Histologically, the degeneration grades of the punctured discs in the OP-1-injected group were significantly lower than those of the lactose-injected group. Biochemically, the PG content of the AF and NP was higher in the OP-1-injected discs than in the control group treated with lactose injection. This study demonstrated the feasibility of restoring degenerative rabbit discs by a single injection of OP-1 into the NP. In addition, the restoration of disc height, which usually began at 6 weeks, was sustained for up to 24 weeks. 

Chondroitinase ABC (C-ABC) chemonucleolysis was used by several authors to simulate an animal model of disc degeneration in the goat disc [61, 62] and in the rat tail [63, 64], and was used also as an alternative degradation method to chymopapain treatment. The effect of OP-1 has been also validated in a chondroitinase-induced chemonucleolysis model in rabbits. These data suggest the feasibility of OP-1 administration in patients with IVD degeneration who had previously received chemonucleolysis that resulted in effective loss of disc height.

Moreover, regarding the stimulatory capacity on matrix accumulation and/or synthesis induced by OP-1, an injection of rhOP-1 performed following chemonucleolysis with C-ABC may be a successful strategy to oppose the degradative effects of the enzyme and to induce the healing of the IVD structure. In adolescent rabbits, C-ABC (10 mU) was first injected into IVDs to induce chemonucleolytic effects [65]. Four weeks after the injection of C-ABC, OP-1 (100 *μ*g/disc) or vehicle was injected and the disc height was measured for up to 12 weeks after the OP-1 treatment. The disc height after the injection of C-ABC was significantly decreased (approximately 34%), and this finding was statistically significant. The injection of OP-1 induced a recovery of the disc height towards normal within 4 weeks after the rhOP-1 injection, gradually approaching the control level by 6 weeks. This change was sustained for up to 16 weeks. The results of an injection of rhGDF-5 in the rabbit anular-puncture IVD degeneration model also confirmed the efficacy of injection of growth ad differentiation factor. The IVDs were punctured by a needle as described in the OP-1 experiment to produce IVD degeneration. Four weeks after, the rabbits received a single injection of rhGDF-5 (10 ng, 1 *μ*g, and 100 *μ*g) or phosphate buffered saline (PBS) and were then followed up for 16 weeks for disc height with MRI findings, and histological grades with biopsy. The injection of rhGDF-5 resulted in a replacement of IVD height and improvements in MRI findings and histological grading scores with statistical significance (*P* < 0.05 − 0.001) [45]. 

In 2-year-old rabbits, the intradiscal injection of rhGDF-5 induced a significant recovery of disc height during a 12-week observation period [45]. The response to the GDF-5 injection was faster than that seen in the adolescent rabbits. Two weeks after the injection, the percent disc height index (%DHI) in the rhGDF-5-injected discs was significantly higher than that in the PBS-injected discs. By 8 weeks afterinjection, the rhGDF-5-injected discs reached the DHI level of the nonpunctured control discs. At 12 weeks after the injection, at MRI the signal intensity in the NP of the rhGDF-5-injected discs was significantly higher than that of the PBS-injected discs. Biomechanically, the viscous and elastic moduli of the IVDs in the rhGDF-5-injected discs were significantly higher than those in the PBS-injected discs. These results indicated the effectiveness of an injection of rhGDF-5 in restoring the degenerative disc in the 2-year-old rabbit anular-puncture disc degeneration model, despite the concern that age-related changes in cell activity might affect the efficacy of growth factor injection therapy [45]. 

In addition, normal discs from the adolescent rabbits (5-6 months) used in these studies still have a large number of notochordal cells [42, 66]. Notochordal cells in the NP were substituted with fibrochondrocytes after the needle puncture, and this difference in cell populations needs to be taken in account for preclinical information in future human clinical trials. 

An age-related decrease in PG and water content and increased degenerative signs in sagittal MRI scans was found in a cross-sectional study using 1–4-year-old rabbits [67]. One can speculate that mature or older rabbits also show less diffusion through the EP because of decreased vascularity, compared to adolescent animals. Therefore, repair of the degenerated IVD may be age-dependent, and mature rabbits may not be able to restore disc structure by growth factor injection as demonstrated in the young animals described above.

Several possible mechanisms for the long-term effects of growth factor injection warrant further investigation. First, the half-life of an injected protein in discs has been considered to be short, in the order of minutes [68]. Finally, the biological and metabolic changes found in the cells following a single injection of a growth factor might be sustained and thus could induce long-term changes in disc structure. However, the results from the use of other GFs have shown some discrepancies in the studies found in the literature. 

Huang et al. injected saline (100 *μ*L) or BMP-2 alone or BMP-2 with coral grafts, after a full-thickness anular tear, in rabbit discs [69]. Radiographs after the treatments revealed that degenerative changes were more frequent and severe in the animals treated with rhBMP-2 with or without the use of coral. At histology, rhBMP-2 promoted fibroblast proliferation and hypervascularity of the intervertebral disc after an anular tear. These discrepancies may result from the fact that the annular tear model simulates an acute condition, and the application of BMP-2 was performed during an acute phase. 

Nagae et al. recently reported findings on the effects of PRP in the rabbit IVD following nucleotomy [70]. PRP was injected into the NP of degenerated IVDs after impregnation into gelatin hydrogel microspheres to produce a slow condition to release the biological factors found in PRP while other IVDs received PRP or PBS without impregnation into gelatine hydrogel microspheres. Gelatin hydrogel immobilize PRP growth factors physicochemically, allowing to release them in a sustained manner with the subsequent degradation of the microspheres. The progression of IVD degeneration was suppressed in the group injected with PRP-microspheres, compared to the PBS and PRP only groups. This finding confirmed Walsh's indication [57] about the necessity to find a sustained delivery system for growth factor injection, which must be specific and dependent on the kind of growth factor injected. In addition, when the defect in the NP is associated with its pathogenesis, such as postdiscectomy, the use of such a scaffold or delivery system may provide a benefit to patents. Some authors found that the adenovirus is an effective vehicle for gene delivery with rapid and prolonged expression of target protein and resulting improvement in markers of disc degeneration. Ad-GDF5 or DMP-7 gene therapy could restore the functions of injured discs and has the potential to be an effective treatment [71, 72].

### 3.5. Limitations of Growth Factor Injection Therapy

There are several limitations of GF injection therapy for IVD degeneration. The cell number is substantially decreased in degenerated discs, especially in the advanced stages [24]. Therefore, a reduced number of cells are present in IVD degenerated IVDs, and only few of them are able to respond to GFs stimulation. Some authors reported specifics methods for prevention of this cellular deficit, such as the association of GFs injection and transplantation of healthy functional cells (i.e., autologous NP cells) [73–76]. Other authors utilized GFs and cells treated with transfection of a therapeutic gene, to obtain the combination effect on the implementation of cells number and the gene therapeutic effect [77].

Nutrition is another important factor linked with the pathogenesis of disc degenerative disease [78]. The IVD is the biggest avascular tissue in the human body, and its survival depends on nutrients diffusion from the vertebral bodies through the EPs. In the degenerated IVD, the sclerosis of the EP did not guarantee a correct diffusion of nutrients, and this condition worsens with a substantial alteration of the IVD tissues and with the worsening of IVD tissues condition and onset of an irreversible state. The increased demand for energy resulting from the stimulation by GFs or by cell supplementation may affect cell viability under those conditions where nutrient transport is compromised. Further investigations of the optimal environment for GF stimulation should be pursued. 

Age-dependent limitations emerged in the therapy of IVD degeneration with GFs, especially concerning the possibility of the few cells present in the degenerated IVD, to respond to GFs stimulation [15]. Because of the emerging trend to treat disc degeneration in adults or older individuals by local injection of growth factors, degeneration stage-related characteristics and age-related findings, changes, and differences in the response to growth factors, are of particular interest for future studies [79].

Often, a delayed response in the recovery of disc height by IVD cells *in vivo* after administration of GFs is observed. The mechanism of this phenomenon remains undetermined. However, it is possible that the half-life of the injected GF may be longer than expected; the capacity to respond to a single administration of a growth factor can continue over a long period of time. Finally, the stimulation by growth factor may determine a cascade of events, eventually resulting in the healing and recovery of disc height.

The *in vitro* and *in vivo* evidence described above supports the hypothesis that the direct injection of GFs into the NP or the AF may be clinically effective as a new therapeutic strategy for the prevention and treatment of IVD degeneration. 

The acceleration of the biological repair process by the stimulation of the cellular anabolic capacity will determine a new category of therapy, where no active treatment currently exists, between conservative therapies and more aggressive therapies such as surgical intervention of vertebral arthrodesis or disc replacement.

## 4. Anticatabolic Agents

The inhibition of degradative molecules can be important to prevent IVD degeneration. Anticatabolics prevent matrix degradation by inhibiting particular enzymes within the disc [20]. Several families of enzymes are capable of breaking down the various molecular components of IVD matrix, including cathepsins, aggrecanases, and MMPs [18, 80]. MMPs play an important role in the normal turnover of matrix molecules, but they have also been linked with degradation of collagen, aggrecan, versican, and link proteins found in the degenerated disc [80]. The main members of the MMP family are collagenase (MMPs 1, 8, and 13), gelatinase (MMPs 2 and 9), and stromelysin (MMP 3). Collagenase and gelatinase cooperate in the breakdown and degradation of collagen contained in the AF. Stromelysin acts prevalently in the NP, and it is involved in the breakdown of the core protein of proteoglycans. Stromelysin is able to access the proteolytic cleavage sites, saving hyaluronate-binding regions, degraded proteoglycan aggregates, and glycosaminoglycan fragments as breakdown products [18, 80–82]. Moreover, TNF-alpha-stimulated gene-6 (TSG-6), found in herniated disks, is another potential therapeutic molecule with anti-inflammatory properties which blocks destructive proteinases [83]. 

In addition, following the current information of synthesis and degradation of IVD tissues, the rate of disc-matrix biological metabolism may also be important. For example, the overall rate of disc metabolism in discs from younger individuals is higher than in discs from older individuals. Moreover, in discs from older individuals, aging promotes the accumulation of degraded products in comparison to the newly synthesized molecules, inducing a catabolic permanent state. This disparity of findings is evident for aggrecans when looking at aged IVDs. Aggrecan and versican degradation is usually performed by another family of metalloproteinases, the ADAMs. Two members of this family (ADAM-TS4 and TS5) show a particular affinity for aggrecan, and are also called aggrecanases [16].

Given the degradative properties of MMPs, more attention should be given to inhibiting MMPs methods, to try to slow or arrest IVD degeneration. MMP activity is physiologically inhibited by tissue inhibitors of MMPs (TIMPs) [82, 84]. Wallach et al. [84] successfully delivered an anticatabolic gene TIMP-I, using an adenoviral vector, into the cells from degenerated IVD. They also demonstrated an increased expression of TIMP-1 in disc cells and also an increase in the proteoglycans contennt. In other studies, TIMP inhibited the breakdown of proteoglycans, such as aggrecan, and may be a potential molecular therapy strategy for the management of IVD degeneration [85, 86].

Finally, cytokines such as TNF-alpha and IL-1 may have critical roles in disc metabolism and the pathogenesis of IVD degeneration. Several inflammatory cytokine antagonists, including IL-1 antagonists and TNF-a antagonists, have also been studied with *in vitro* and *in vivo* [87].

IL-1Ra and Infliximab, which can reduce IL-1 and TNF-alpha blood levels, respectively, may be also useful as an alternative pharmacological therapy for the IVD degeneration [88–90]. In addition, recent studies demonstrated the possibility to reduce IVD degeneration by the inhibition of TNF-alpha molecules using etanercept. Perispinal etanercept injection may be useful for both acute and chronic disc-related pain, especially in patients with chronic refractory disc-related pain [91]. Recently, Horii et al. [92] conducted an interesting experiment using etanercept in the dorsal root ganglia (DRG) of rats. The neurotracer FluoroGold was applied to the surfaces of L4/5 IVD to label their innervating DRG neurons (*n* = 30). Of 30 rats, 10 were in a nonpunctured disc sham surgery control group whereas the other 20 were in experimental groups in which intervertebral IVDs were punctured with a 23G needle. Etanercept or saline was applied into the punctured discs (*n* = 10 each treatment). After 14 days of surgery, DRGs from L1 to L6 were harvested, sectioned, and immunostained for CGRP. The proportion of FluoroGold-labeled CGRP-immunoreactive DRG neurons was evaluated in all groups. CGRP was upregulated in DRG neurons innervating damaged discs. However, direct intradiscal application of etanercept immediately after disc puncture suppressed CGRP expression in DRG neurons innervating injured discs. In a double-blind, placebo-controlled, dose-response pilot study, Cohen et al. [93] reported that no serious side effects were observed after a single low-dose intradiscal injection of etanercept. The same conclusions were reached in a blind randomized controlled trial using etanercept injection for the treatment of sciatica [56]. The study [56] did not show benefits after the use of etanercept over placebo in the pharmacologic treatment of sciatica. 

Finally, CPA-926 can be used in the IVD degeneration for its anti-inflammatory and anti-tumorigenic properties. Following oral administration of CPA-926, histological and radiographic findings on an annulotomy model of IVD degeneration in rabbits showed a delay of the onset of disc-height loss [94]. Further research into anticatabolic molecules should be performed.

### 4.1. Limitations of Anticatabolic Therapy

The most important limitation of this approach is linked to the chronic nature of the IVD degeneration disorder. IVD degeneration is a long-term problem, occurring gradually and worsening with aging. The normal *in vivo* half-life found for these therapeutic molecules is on the order of only few minutes, and this suggests that the effects of these molecules will not last for significant periods of time. Therefore, if anticatabolic therapy can be effective in the long term, repeat or continuous infusions need to be performed, limiting the clinical utility of direct molecular-based therapies [95–107].

## 5. Summary

The normal intervertebral disc is a complex structure able to dissipate loads and permit multiaxial motion of the spine. The homeostasis of the IVD tissues is maintained trough a fine balance between matrix synthesis and degradation, which determines biological changes and microstructural modification and consequent organization. IVD degeneration leads to the alteration and loss of function of the IVD tissues, disrupting the well-defined organization of IVD structures and also of the IVD biomechanical balance. The major problem of IVD degeneration is the progressive loss of proteoglycan and water in the NP, followed by matrix degeneration and presence of catabolic molecules in the IVD tissues [108–114]. The restoration of the proteoglycans content in the NP, such as the inhibition of the molecules involved in the matrix degeneration including, can be a possible solution for management of IVD degeneration. Several biological molecules can be potentially useful and effective in IVD repair. GFs and anticatabolic molecules are possible fields of study and intervention. Many of these molecules have been investigated *in vitro*, and several have been used in animal model of IVD degeneration. The next step of research should be performed with animal model of IVD degeneration to better simulate the human condition. In addition, the use of GFs and of anticatabolic molecules demonstrated important limitations for future preclinical studies [6, 7, 20, 53, 114–163].

Major drawbacks in the field include the poor delivery methods, the rapid effect and formulation of therapeutic molecules, and the lower percentage of cells able to respond to GFs and anticatabolic stimulation found in degenerated IVD tissues. GFs injection can be useful in IVD degeneration if administered in the early phase of the disease while the reduced number of cells found in IVD degenerated tissues can be a major problem [95, 104, 105, 164–188]. Only few cells are present in IVD degenerated tissues and are able to respond to GFs stimulation. Anticatabolic molecules show a very short-term effect and the *in vivo* half-life found of these therapeutic molecules is on the order of only few minutes suggesting that the effects of these molecules will not last for significant periods of time. Future research should focus on biological therapy research for the treatment of IVD degeneration.
